# FGF2 and EGF for the Regeneration of Tympanic Membrane: A Systematic Review

**DOI:** 10.1155/2021/2366291

**Published:** 2021-06-29

**Authors:** Zhengcai Lou, Zihan Lou, Yumeng Jiang, Zhengnong Chen

**Affiliations:** ^1^Department of Otorhinolaryngology, Yiwu Central Hospital, Yiwu City, 322000 Zhejiang Province, China; ^2^Department of Otolaryngology-Head and Neck Surgery, Shanghai Jiao Tong University Affiliated Sixth People's Hospital, Shanghai 200233, China; ^3^Otolaryngology Institute of Shanghai Jiao Tong University, Shanghai 200233, China

## Abstract

**Objective:**

A systematic review was conducted to compare the effectiveness and safety of fibroblast growth factor-2 (FGF2) and epidermal growth factor (EGF) for regeneration of the tympanic membrane (TM).

**Methods:**

The PubMed database was searched for relevant studies. Experimental and clinical studies reporting acute and chronic TM perforations in relation to two healing outcomes (success rate and closure time) and complications were selected.

**Results:**

A total of 47 studies were included. Five experimental studies showed closure rates of 55%–100% with FGF2 compared with 10%–62.5% in controls for acute perforations. Five experimental studies showed closure rates of 30.3%–100% with EGF and 3.6%–41% in controls for chronic perforations. Two experimental studies showed closure rates of 31.6% or 85.7% with FGF2 and 15.8% or 100% with EGF. Nine clinical studies of acute large perforations showed closure rates of 91.4%–100% with FGF2 or EGF. Two clinical studies showed similar closure rates between groups treated with FGF2 and EGF. Seven clinical studies showed closure rates of 88.9%–100% within 3 months and 58%–66% within 12 months using FGF2 in repair of chronic perforations, but only one study showed a significantly higher closure rate in the saline group compared with the FGF2 group (71.4% vs. 57.5%, respectively, *P* = 0.547). In addition, three experimental studies showed no ototoxicity associated with FGF2 or EGF. No middle ear cholesteatoma or epithelial pearls were reported, except in one experimental study and one clinical study, respectively.

**Conclusions:**

FGF2 and EGF showed good effects and reliable safety for the regeneration of TM. In addition, EGF was better for the regeneration of acute perforations, while FGF2 combined with biological scaffolds was superior to EGF for chronic perforations, but was associated with high rates of reperforation over time. Further studies are required to determine whether EGF or FGF2 is better for TM regeneration.

## 1. Introduction

Tympanic membrane (TM) perforation is a common entity encountered in otology clinics, which results in hearing loss, recurrent middle ear infections, changes in lifestyle, and risk of cholesteatoma formation. Most acute perforations tend to heal spontaneously. However, a few acute perforations and most chronic perforations fail to heal and require myringoplasty. Commonly used graft materials include autologous fascia, fat, perichondrium, and cartilage. Some biological materials have been developed for use in myringoplasty, e.g., bacterial membranes [1], hyaluronic acid [2], growth factors [3], and acellular collagen scaffolds (ACSs) [4]. Fibroblast growth factor-2 (FGF2) and epidermal growth factor (EGF) are the most common growth factors used in wound repair [4, 5]. EGF is a single-chain polypeptide chain of 53 amino acids first isolated from the submaxillary glands of mice [6], which stimulates epidermal cell proliferation and keratinization both *in vitro* and *in vivo*. FGF2 is a 146-amino acid polypeptide initiator of mesoderm- and ectoderm-derived cells, including fibroblasts, endothelial cells, and epithelial cells [6]. Enhanced wound repair in skin has been demonstrated after application of growth factors [7]. In addition, EGF and FGF2 have also been applied to repair brain neuron damage [8], corneal injury [9], and facial nerve injury [10] and to promote scarless healing [11]. Some recent studies have demonstrated that FGF2 and EGF are good candidates for TM regeneration because they both act on epithelial cells and fibroblasts that are involved in TM repair [12, 13], and both clinical and experimental studies yielded encouraging results [14–55]. However, whether EGF or FGF2 is better for TM regeneration remains unclear. This study is aimed at reviewing systematically the healing outcome and side effects of EGF and FGF2 on the TM regeneration.

## 2. Materials and Methods

This study followed the Population, Intervention, Comparison, Outcome (PICO) format. The study question was as follows: For people with TM perforations, can the use of FGF2 or EGF improve both the healing rate and time and hearing outcomes? This review was performed in accordance with the Preferred Reporting Items for Systematic Reviews.

### 2.1. Search Strategy and Study Selection

**Figure 1:**
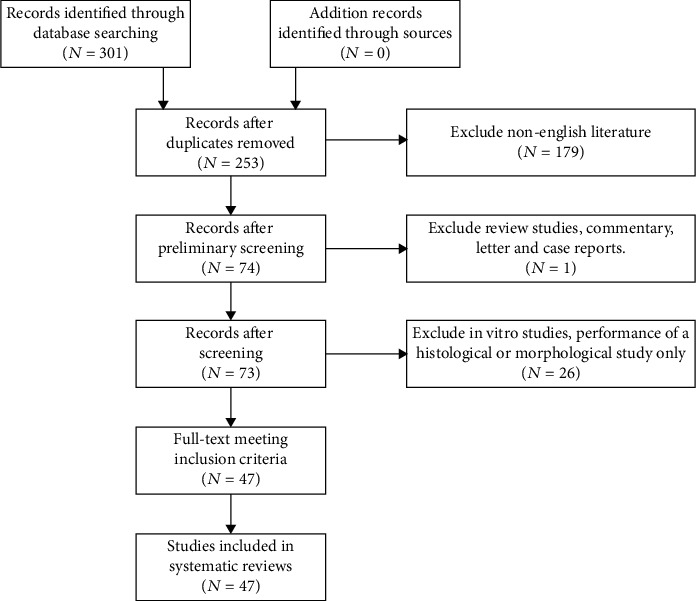
Flow diagram of the search process and search outcomes.

A comprehensive search of the literature was conducted using the PubMed (US National Library of Medicine) database from establishment to January 30, 2021. The key words used in the search were as follows: tympanic membrane(s), eardrum(s) or tympanic membrane perforation(s), eardrum perforation(s), tympanic membrane rupture, eardrum rupture, fibroblast growth factor-2, basic fibroblast growth factor, epidermal growth factor, heparin-binding growth factor, HFGF2-2, HB-EGF, and collagen-binding FGF2. The original articles were all from peer-reviewed scientific journals published in English (Figure 1).

### 2.2. Inclusion and Exclusion Criteria

The inclusion criteria were as follows: observational studies (retrospective or prospective) or treatment studies (randomized controlled trials [RCTs]), studies that reported the outcomes of application of FGF2 and EGF in adult or pediatric populations, and animal studies with healing outcomes (closure rate and/or closure time). The exclusion criteria were as follows: histological or morphological study only, *in vitro* studies, review studies, commentary, letters, and case reports.

The titles and abstracts were screened independently by two researchers to identify potentially relevant articles, and the full-text articles were then retrieved. The bibliography of each article was also searched for further potentially relevant studies. All articles that met the inclusion criteria were reviewed for data extraction and quality assessment.

### 2.3. Definition of Acute and Chronic Perforations

Acute perforation was defined as sunderly rupture of the TM due to a rapid change in atmospheric pressure (including barotrauma, slap to the ear, or blast injury), penetrating injury, or incision injury. Chronic perforation was defined as rupture due to trauma and chronic otitis media (COM) that failed to heal within 3 months [56–58].

### 2.4. Outcome Measures

The primary outcome measure was the complete closure rate in the FGF2 or EGF treatment group compared to the complete closure rate in the control group. The secondary outcomes were the differences in healing time and improvement in hearing. The following data were obtained or derived from the full reports of the 47 studies for both the treatment and control groups: number of subjects, percentage closure, and mean and standard deviation (SD) of closure time in days. We also recorded the first author, year of publication, the study design (RCT or non-RCT), and size of perforations targeted by the study.

## 3. Results

A total of 73 articles were initially retrieved in the search. However, 26 articles did not meet the inclusion criteria, and only the remaining 47 articles were included in the analysis. Of the 47 papers, 18 were experimental studies of the effects of FGF2 or EGF in repair of acute or chronic perforations [13–30] (Table 1), 16 were clinical studies in human acute perforations (FGF2 in 11 and EGF in 5) [31–46] (Table 2), nine were clinical studies in human chronic perforations (FGF2 in eight and EGF in one) [47–55] (Table 3), and four papers examined the dose- and time-dependent effects of FGF2 or EGF on human and experimental perforations [56–59] (Table 4).

The 11 clinical studies of the effects of FGF2 on acute perforations were from China [31–41]; four studies were randomized controlled trials (RCTs), six were prospective studies, and the remaining one was a retrospective study. Of the eight clinical studies of the effects of FGF2 on chronic perforations, seven were from Japan [47–53] and only one was from the USA [54]. Two studies were randomized controlled trials (RCTs), five were prospective studies, and the remaining one was a retrospective study. Of the eight animal studies of the effects of FGF2 on acute perforations, eight were prospective studies. All three of the animal studies of the effects of FGF2 on chronic perforations were prospective studies.

Five clinical studies of the effects of EGF on acute perforations were from China [42–46]; two were randomized controlled trials (RCTs), two were prospective studies, and the remaining one was a retrospective study. Only one RCT of the effects of EGF on the regeneration of human chronic perforations was found. Both of the animal studies of the effects of EGF on acute perforations and all of five animal studies of the effects of EGF on chronic perforations were prospective.

### 3.1. Treatment Technique

**Figure 2:**
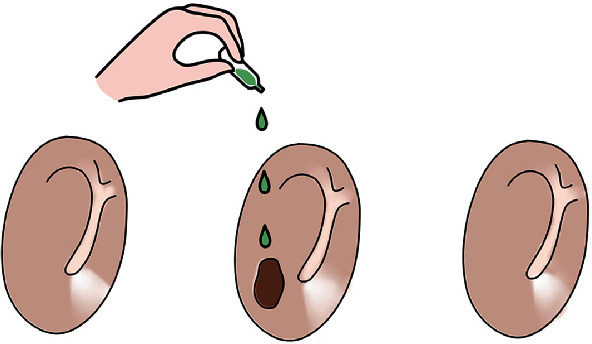
Diagram of EGF or FGF2 in repair of acute perforation.

**Figure 3:**
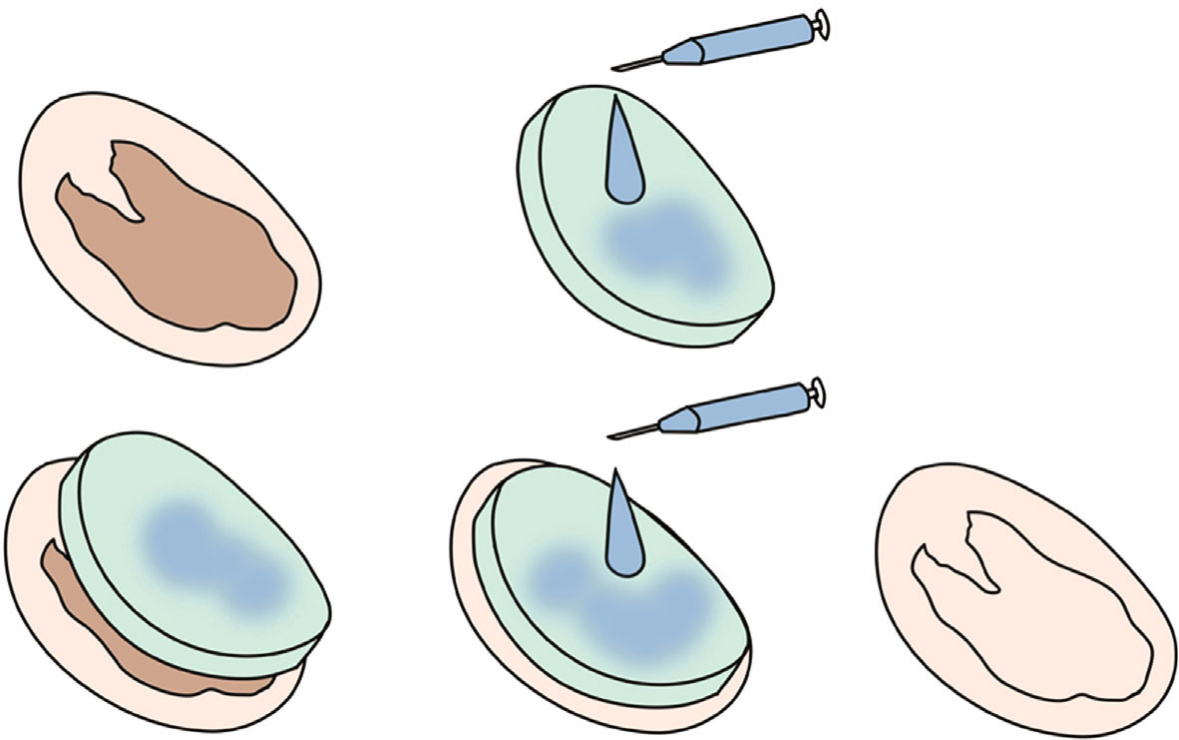
Diagram of EGF or FGF2 in repair of chronic perforation.

The TM was treated by the direct application of FGF2 or EGF alone or combined with Gelfoam in all of the clinical studies for acute perforations (Figure 2). However, the TM was repaired by FGF2 or EGF via biological scaffold for chronic perforations (Figure 3).

### 3.2. Healing Outcomes of FGF2 or EGF for Repairing TM Perforations

#### 3.2.1. FGF2 or EGF in Repair of Experimental Acute Perforations

Of the six studies evaluating the healing outcomes of FGF2 combined with biological scaffold (including Gelfoam, glycerol, and ACS) on acute perforations, one study showed the same healing rate of 100% for FGF2 and stabilizer solvent combined with glycerol [14], and two studies found that the closure rate in the FGF2 group combined with Gelfoam was significantly higher than that of the control group (including PBS, buffer solution) (55%–100% vs. 0%–41%, respectively, *P* < 0.001) [13, 20]. Vrabec et al. [15] found a significant difference in the average healing time associated with use of FGF2 combined with glycerol vs. glycerol alone (9.74 ± 2.31 vs. 13.74 ± 4.93 days, respectively, *P* < 0.001). However, one study by Zhang et al. [21] showed that, although the closure rate associated with use of FGF2 combined with collagen-binding domain was high compared with collagen membrane on day 14, the difference was not significant (100% vs. 75%, respectively, *P* = 673). Yao et al. [23] also reported that the differences in closure rates were not significant between ACS, bFGF, and ACS+bFGF at 2 weeks (100% vs. 100% vs. 100%, respectively, *P* = 0.841), but closure rates were high with ACS alone or combined with FGF2 compared with FGF2 alone (71.4% vs. 100% vs. 42.9%, respectively, *P* < 0.001).

Only two studies compared the healing outcomes of FGF2 alone and other solutions, including stabilizer solvent or sterile saline. One study by Fina et al. [14] showed encouraging results and reported closure rates of 60% with FGF2 alone and 30% with stabilizer solvent alone by 7 days for 1 mm perforations and 100% with FGF2 alone and 33% with stabilizer solvent alone by 14 days for 2 mm perforations. However, another study by Friedman et al. [17] reported similar closure rates of 100% with FGF2 alone and with sterile saline alone.

Two studies described use of EGF combined with Gelfoam for repairing acute or subacute perforations. Ramalho and Bento [26] reported closure rates of 30.3% with EGF, 3.6% with pentoxifylline, and 16.5% with EGF+pentoxifylline for 30 days. Güneri et al. [25] reported that the difference in average healing time between hyaluronic acid and EGF groups was not significant (8.8 ± 1.6 and 7.4 ± 1.6 days, respectively, *P* > 0.05) but was significantly shortened compared with spontaneous healing (15 ± 2 days, *P* < 0.01).

#### 3.2.2. FGF2 or EGF in Repair of Experimental Chronic Perforations

Two studies evaluated the efficacy of FGF2 in repair of chronic perforations. Kato and Jackler [16] reported a closure rate of 81% by 4 weeks associated with FGF2 combined with Gelfoam compared to 41% by 6.5 weeks associated with buffer solution combined with Gelfoam, while Ozkaptan et al. [18] showed closure rates of 86.7% (13/15) with FGF2 alone and 13.3% (2/15) with saline solution alone at 20 days. These results suggested that FGF2 with or without biological scaffold was associated with a significantly higher closure rate compared with saline solution in repair of experimental chronic perforations.

Five studies examined the efficacy of EGF combined with biological scaffold (including Gelfoam, chitosan patch, and polymer) for repair of chronic perforations, four of which showed that the closure rate in the EGF group was significantly higher than the control group (56.5%–100% vs. 20%–41%, respectively) [24, 27, 28, 30]; in the remaining study by Dvorak et al. [29], the results indicated similar closure rates between EGF and PBS control groups (100% vs. 80%, respectively, *P* = 0.873). However, there have been few studies of the effects of EGF alone in repair of chronic perforations. Nevertheless, these studies provided encouraging results regarding the use of EGF in repair of chronic perforations. In addition, the application time taken to reach a similar closure rate was 3–4 weeks for FGF2 [16, 18] but 4–10 weeks for EGF [24, 27–30].

#### 3.2.3. FGF2 or EGF in Repair of Human Acute Perforations

All of nine clinical studies of FGF2 in repair of acute perforations identified in the literature search were performed by Lou et al. [31–37, 40, 41]. Three clinical studies showed that FGF2 with or without Gelfoam patching significantly improved the closure rate (91.7%–100% vs. 52.9%–77%, respectively) and shortened the closure time compared with spontaneous healing for large perforations [32, 34, 36]. Although the difference in closure rate was not significant for medium-sized perforations (95.5%–98.5% vs. 73.4%–89.8%, respectively), the average closure time associated with use of FGF2 was significantly shortened compared to that of spontaneous healing in three studies [31, 40, 41]. In addition, Lou et al. [35] performed a prospective clinical study of FGF2 on blast-induced subtotal perforations and reported a closure rate of 94.1% with an average closure time of 28.4 ± 10.9 days. In addition, FGF2 alone significantly shortened the closure time compared with spontaneous healing for penetrating perforations (12.6 ± 1.2 vs. 43.1 ± 2.5 days, respectively, *P* < 0.01), although the difference in closure rate was not significant (100% vs. 77%, respectively, *P* < 0.001) [33]. Nevertheless, a prospective controlled study of FGF2 alone, 0.3% ofloxacin eardrops, and Gelfoam patching in cases of medium and large perforations showed that there were no differences in closure rate (93.2% vs. 85.7% vs. 92.3%, respectively, *P* = 0.257) or average closure time (12.3 ± 8.15 vs. 14.3 ± 5.44 vs. 13.97 ± 8.82 days, respectively, *P* < 0.001) between treatments [37].

Lou et al.'s institution also performed clinical studies of EGF in the repair of acute perforations and reported that EGF alone significantly improved the closure rate (91.4%–96.2% vs. 61.1%–85.2%, respectively) and shortened the closure time (8.9 ± 2.3 vs. 24.6 ± 9.7 days and 9.1 ± 3.9 vs. 20.6 ± 10.7 days) compared with spontaneous healing, with an average shortening of closure time by 2 weeks [42, 45]. However, no differences were found between EGF alone and 0.3% ofloxacin eardrops in closure rate (93.5% vs. 93.2%, respectively, *P* = 0.19) or closure time (12.9 ± 5.3 vs. 13.3 ± 4.9 days, respectively, *P* = 0.84) [43]. In addition, there were no significant differences between EGF alone and Gelfoam patching in closure rate (97.8% vs. 86.7%, respectively, *P* = 0.039) or average closure time (11.12 ± 4.60 vs. 13.67 ± 5.379 days, *P* = 0.071) [44]. However, Lou [46] used EGF alone to treat 24 adult chronic traumatic perforations and reported a closure rate of 100% within 6.1 ± 2.3 days.

#### 3.2.4. FGF2 or EGF in Repair of Human Chronic Perforations

Of the eight clinical studies of FGF2 combined with biological scaffold in repair of chronic perforations, there were no reports of the application of FGF2 alone. In two case controlled studies, the FGF2 group showed significant improvement in the closure rate compared with controls (including saline via atelocollagen or Gelfoam) (98.1% and 100% vs. 10% and 40%, respectively) [47, 49]. In another five case observation studies or retrospective cohort studies, the closure rates were 58%–92% [48, 50–53] and tended to decrease over time (88.9%–100% and 58%–66% at 3 and 12 months posttreatment, respectively) [48–53]. In contrast, Santos et al. [54] reported a higher closure rate in the saline control group compared with the FGF2 group (71.4% vs. 57.5%, respectively, *P* = 0.547). Traumatic and ventilation tube- (VT-) induced perforations were included in five studies [50–54]. However, Ramsay et al. [55] reported a randomized control trial of EGF in repair of chronic perforations and found healing in only one case in the PBS group and in no cases in the EGF group.

### 3.3. Comparative Studies of the Effects of FGF2 and EGF on TM Regeneration

Only two experimental studies compared the efficacy of FGF2 and EGF in the healing of TM perforations. Chauvin et al. [19] reported closure rates of 100% (7/7) with EGF on day 21 and 85.7% (6/7) with bFGF on day 32 in the repair of acute perforations. However, Santa Maria et al. [22] used FGF2 and EGF to repair chronic perforations in a mouse model and reported closure rates of 31.6% (6/19) with FGF2 and 15.8% (3/19) with EGF, but closure rate reached 83.3% in the HB-EGF group. In addition, two clinical studies comparing the efficacy of FGF2 and EGF in repair of acute perforations were performed by the same authors, and they reported similar closure rates and average healing times between FGF2 and EGF (89.3% vs. 86.2% and 93.18% vs. 91.11%) [38, 39]. However, the literature search identified no clinical comparative studies of FGF2 and EGF in repair of chronic perforations.

### 3.4. Dose and Time Dependency of the Effects of FGF2 or EGF on TM Regeneration

Mondain et al. [60] compared the efficacies of different dosages of FGF2 on the regeneration of acute perforations. The reported healing rates were 100% within 3.16 days in 2000 ng, 80% (12/15) within 6.1 days in 400 ng, and 60% (9/15) within 6.3 days in 200 ng, but the high dosage of 2000 ng caused myringitis and hyperplasia of the external auditory canal (EAC). Lou et al. [35] compared the efficacies of high and low dosages of FGF2 repairing human acute perforations; they found that, although the closure rate was similar between the two groups (100% vs. 92%, respectively, *P* = 0.597), the low dosage of FGF2 significantly shortened the average closure time compared with the high dosage (10.20 ± 5.13 vs. 14.39 ± 6.20 days, respectively, *P* < 0.001). Lou et al. [45] reported similar results with EGF (10.20 ± 5.13 vs. 14.39 ± 6.20, *P* < 0.001). In addition, the clinical study performed by Lou et al. also showed that delayed application of FGF2 or EGF resulted in a shorter average closure time compared with early application (8.5 ± 2.1 vs. 17.5 ± 5.1 days, respectively, *P* < 0.001 and 11.25 ± 7.15 vs. 13.15 ± 5.80 days, respectively, *P* < 0.001) [57, 59].

### 3.5. Side Effects of FGF2 or EGF in TM Regeneration

All clinical studies showed that application of FGF2 or EGF did not affect hearing improvement [31–55]. In contrast, Lou et al. [35] reported that audiometry improved significantly after treatment with FGF2 alone for TM perforations due to blast injury. Yao et al. [23] reported that the hearing recovery in the FGF2 group was faster compared to spontaneous healing based on auditory brainstem response (ABR). Santa Maria et al. [22] reported that there was no difference in hearing between EGF-treated and control ears regardless of ABR or distortion product otoacoustic emission score. Kase et al. [59] examined the ototoxicity of FGF2 and observed no differences in cochlear potential or hair cell structure between FGF2 treatment and control groups. Lee et al. [28] reported no significant pathology in surface preparations of the organ of Corti after EGF treatment.

Although middle ear cholesteatoma was mentioned in three experimental studies [17, 23, 60], there was no direct evidence that FGF2 induced cholesteatoma. Only Dvorak et al. [29] reported two intratympanic pearls and a middle ear cholesteatoma in ears treated with EGF in their experimental study. In addition, none of the clinical studies described any cases of middle ear cholesteatoma after topical application of FGF2 or EGF. Lou et al. [41] reported that temporal bone computed tomography (CT) revealed pneumatolytic middle ear and mastoid cells during 2-year follow-up after TM repair with FGF2 treatment. However, Hakuba et al. [50] reported epithelial pearl formation following FGF2 treatment.

## 4. Discussion

### 4.1. Bioactivity and Delivery of FGF2 and EGF

It is well known that EGF and FGF2 play major roles in wound healing, and both have been used as regeneration factors in a diverse range of conditions, including burns, chronic wounds, oral ulcers, vascular ulcers, diabetic ulcers, pressure ulcers, and surgical incisions [8–11, 61–63]. Growth factors trigger specific target cells by binding to their high-affinity surface membrane receptors. Once such factors have bound to the target cell surface receptor, and the target cells are then activated to undergo mitosis or chemotaxis, thereby promoting the proliferation and chemotactic migration of target cells and neovascularization, thus improving wound healing. EGF induces the proliferation of epithelial cells, endothelial cells, fibroblasts, and keratinocytes, but mainly stimulates chemotactic migration and is an effective mitogen for epithelial cells [4–6]. FGF2 is chemotactic and an effective mitogen for vascular endothelial cells and fibroblasts, and application of FGF2 has been shown to increase connective tissue and granulation tissue formation [4–6]. Nevertheless, the *in vitro* half-life is approximately 12 hours for FGF2 and 4 hours for EGF at physiological pH and temperature [64, 65]. Therefore, biological scaffolds are usually applied to achieve sustained delivery of EGF or FGF2 for continuous exposure of target cells and thus maintain the biological effects.

### 4.2. Effects of FGF2 and EGF on TM Regeneration

FGF2 and EGF were shown to facilitate regeneration of acute TM perforation [13–15, 20–23, 31–37, 40–42, 45]. However, although most experimental studies showed that FGF2 treatment resulted in significantly higher closure rates compared with PBS or saline solution, three experimental studies indicated the same acute perforation closure rate of 100% for FGF2 and stabilizer solvent [14], sterile saline [17], or HA [19]. Clinical studies showed the same results [37, 39, 43]. These results suggested that FGF2 and EGF appear to have no real advantage compared with ofloxacin eardrops or Gelfoam patching for acute perforations. These observations raise the question of whether the effects of FGF2 or EGF on TM regeneration are due to the biological effects of the growth factors, ambient effects of the moist environment, or synergistic actions of both factors. Some groups have suggested that the moist environment aided TM healing, but an excessively wet environment impaired TM healing [66–68]. Whether was the low healing rate in the control group related to the application of high dosage of solution in some experimental studies? This has also been demonstrated by clinical and experimental studies, which showed that a high dosage of FGF2 or EGF reduced the healing rate and prolonged the healing time [58, 59]. A high dosage of FGF2 was shown to inhibit collagen synthesis in wound repair [69]. Therefore, differences in the dosage and start time of application could have led to inconsistencies in the results between groups.

Experimental studies suggested that FGF2 with or without scaffold facilitated the regeneration of chronic perforations [16, 18, 22]. Similarly, clinical studies also showed encouraging results with regard to FGF2 in the repair of chronic perforations [47–49]. Unfortunately, these clinical studies added biological scaffolds, which can itself close the perforations [1, 2, 4, 70, 71]. In addition, the study populations also included cases of chronic traumatic and VT-induced perforations, which have high spontaneous healing rates and differ from perforations with COM [72, 73]. In addition, it is worth noting that the closure rate tended to decrease with increasing follow-up time [50–52]. The high reperforation rate could be related to impairment of long-term collagen accumulation by continuous FGF2 application [69, 74]. Although EGF provided encouraging results for repairing acute perforations and experimental chronic perforations, only one clinical study of EGF in repair of chronic TM perforations has been reported to date [55], which showed failure of the treatment with only one case showing healing in the PBS group and no cases of healing in the EGF group. Conflicting results were also found regarding FGF2 in repair of human chronic perforations. An RCT of 54 patients with chronic perforations by Santa Maria et al. [22] showed that the closure rate was not significantly different between the saline and the FGF2 treatment group (71.4% vs. 57.5%, respectively, *P* = 0.547). In addition, although experimental studies showed high closure rates of chronic perforations following FGF2 or EGF treatment, these experimental chronic perforations did not correspond to actual chronic perforations with COM, but only acute perforations with delayed healing [56–58]. Therefore, from limited clinical and experimental data, it is difficult to evaluate objectively the efficacy of FGF2 or EGF in the repair of chronic TM perforations, and a great deal of work remains to be done regarding the effects of FGF2 or EGF on regeneration of chronic TM perforations.

### 4.3. Contrasting Effects of FGF2 and EGF in TM Regeneration

Although topical application of exogenous EGF or FGF2 promoted TM regeneration, it remains unclear whether EGF or FGF2 is better for TM regeneration. It is well known that each growth factor has some degree of selectivity with regard to chemotactic activity and mitosis of cells in wound repair. A study of corneal epithelial wound healing indicated that EGF markedly promoted corneal epithelium repair in the short term, while FGF2 did not, but rh-EGF showed weaker promotion of neovascularization (CNV) than FGF2 [75]. Other studies have shown that FGF2 has stronger effects on promoting neovascularization and cell proliferation and oral mucosa ulcer healing than EGF [76, 77]. Similarly, FGF2 predominantly affected the fibrous layer, induced the proliferation of fibroblasts, and regulated the reaction of connective tissue during the TM repair process, whereas EGF stimulated the epithelial layer and promoted the proliferation and migration of epithelial cells and keratinocytes [64, 78].

TM closure can consist of healing of only the epithelial layer but not the fibrous layer, e.g., spontaneous healing of the perforations with COM, and the simultaneous closure of the epithelial layer and fibrous layer or orderly closure of the epithelial layer and fibrous layer, e.g., spontaneous healing of the normal TM. Fibroblasts grow much faster than epithelial cells and can lead to the formation of granulation tissue in the fibrous layer if FGF2 is applied alone, which can prohibit closure of the epidermal layer in some cases [24]. Theoretically, EGF minimizes this problem as it promotes the regeneration of epithelial cells in the outer epidermal layer [24]. This has been demonstrated in clinical studies. Two clinical studies comparing the effects of FGF2 and EGF in repair of acute perforations showed faster closure in the EGF group compared to the FGF2 group [38, 39]. FGF2 has been most frequently used to repair chronic perforations in clinical cases because the fibrous layer comprises 98% of the TM [24]. By light and electron microscopy, Magnuson et al. [73] reported altered collagen structure and disorganized collagen layer in chronic perforations with COM but normal collagen structure in traumatic perforations and showed that the TM remnant with COM had lost part of its normal healing potential. However, FGF2 can wider stimulate the proliferation of fibroblasts and neovascularization in the fibrous layer, thereby recovering the normal collagen structure and facilitating the healing of chronic perforations. Therefore, successful closure can usually be obtained in most cases if the perforation is covered with FGF2 combined with a biological scaffold. That is, EGF showed better healing outcomes than FGF2 for acute perforations, but FGF2 had better healing outcomes compared with EGF for chronic perforations with COM.

### 4.4. Side Effects and Prospects for Clinical Application of FGF2 or EGF in TM Regeneration

The side effects of FGF2 or EGF used in regeneration of TM have been widely investigated in both clinical and experimental studies [13–55]. Application of FGF2 to the external and middle ear does not seem to be associated with any apparent risk of ototoxicity. ABR thresholds indicated that none of the ears treated with EGF or FGF2 showed hearing loss after TM closure. There was no evidence of squamous cell elements or cholesteatoma formation on the medial surface of the TM in ears treated with EGF or FGF2. In contrast, FGF2 was shown to protect spiral ganglion neurons against glutamate neurotoxicity *in vitro* and hair cells from acoustic trauma [79, 80]. Although an experimental study showed that application of high dosage of FGF2 caused myringitis and hyperplasia of the EAC [56], which may be avoided through application of an appropriate dosage by clinic. In addition, although a few groups reported lower incidence rates of intratympanic pearls [29, 50], this was not specific for FGF2 or EGF and may been seen in most types of myringoplasty [81, 82].

Treatment with FGF2 or EGF for TM regeneration is safe, and application of FGF2 or EGF alone significantly facilitated the regeneration of acute perforations. However, FGF2 combined with biological scaffold may be more efficient for chronic perforations with COM. Unfortunately, all clinical studies of FGF2 and EGF in TM repair identified in our literature search were from China and Japan [31–53], and there was only one multicenter clinical study with a small sample size [53]. In addition, continuous release of FGF2 or EGF is required to maintain the biological effects via delivery systems because of their short half-life and growth factor eardrops have limited efficacy. Therefore, further studies to develop better growth factor preparations for use in TM repair are required.

## 5. Conclusions

FGF2 and EGF showed good effects and reliable safety for the regeneration of TM. In addition, EGF was better for the regeneration of acute perforations, while FGF2 combined with biological scaffolds was superior to EGF for chronic perforations, but was associated with high rates of reperforation over time. Further studies are required to determine whether EGF or FGF2 is better for TM regeneration.

## Figures and Tables

**Table 1:** Summary of FGF2 and EGF effects on experimental perforation.

Authors	Study subject	Treatment strategy	Vehicle	Healing outcome
Fina et al. [13] (1991)	GP-acute	1 *μ*g FGF-2 vs. PBS only+Gel	Gel	1 mm TMPs: 55% in FGF-2 vs. 10% in PBS at 3 days; 2 mm TMPs: 87.5% in FGF-2 vs. 0% in PBS at 5 days
Fina et al. [14] (1993)	GP-acute	Group 1: 1 *μ*g FGF-2 vs. 1 *μ*g placebo (stabilizer solvent) alone; group 2: 1 *μ*g FGF-2 vs. 1 *μ*g stabilizers solvent	Group 1: no; group 2: Gel	Group 1. 1 mm TMPs: 60% in FGF-2 vs. 30% in placebo group by 7 days; 2 mm TMPs: 100% in FGF-2 vs. 33% in placebo group by 14 days Group 2. 2 mm TMPs: 100% in FGF-2 vs. 100% in placebo group by 14 days
Vrabec et al. [15] (1994)	Rats-acute	100 *μ*g/ml FGF-2 vs. Gly	Gly	9.74 ± 2.31 days in FGF-2 vs. 13.74 ± 4.93 days in glycerol
Kato & Jackler [16] (1996)	Chinchillas-chronic	FGF-2 vs. buffer solution	Gel	81% by 4 weeks in FGF-2 vs. 41% by 6.5 weeks in buffer solution
Friedman et al. [17] (1997)	Chinchilla-acute	FGF-2 vs. sterile saline for 2 weeks	NO	100% in FGF-2 with 8-12 days vs. 100% in control group 6-18 days
Ozkaptan et al. [18] (1997)	GP-chronic	400 ng FGF-2 vs. saline solution	No	86.7% (13/15) in FGF-2 vs. 13.3% (2/15) in saline solution at 20 days
Chauvin et al. [19] (1999)	GP-acute	1 mg HA, 0.4 *μ*g FGF-2, 1.0 *μ*g EGF vs. 0.1 ml Vasocidin	Vasocidin	100% (7/7) in HA and 100% (7/7) in EGF at day 21, 85.7% (6/7) in FGF-2 and 63.6% (21/33) in Vasocidin at day 32
Hakuba et al. [20] (2014)	GP-acute	FGF-2 vs. saline vs. control (FGF-2 or saline alone)	Gelatin HG	100% in FGF2-HG, 62.5% in saline-HG, and 0% in no HG after 30 days
Zhang et al. [21] (2017)	SD rats-acute	FGF 2 vs. CM vs. SH	CM-CBD	100% (16/16) in CM-CBD-FGF2, 75%(12/16) in CM, and 68.8% (11/16) in SH at day 14
Santa Maria et al. [22] (2015)	Mice-chronic	HB-EGF, FGF-2, EGF, polymer	Polymer	83.3% (15/18) in HB-EGF; 31.6% (6/19) in FGF-2; 15.8% (3/19) in EGF; 27.8% (5/18) in polymer for 4 weeks
Yao et al. (2020) [23]	SD rats-acute	ACS vs. FGF-2 vs. ACS+FGF-2 vs. SP	ACS	At one week: 71.4% vs. 42.9% vs. 100% vs. 0; at 2 weeks: 100% vs. 100% vs. 100% vs. 42.9%
Seonwoo et al. [24] (2013)	SD-chronic	EGF-CPS vs. SH	CPS	56.5% (13/23) vs. 20.8% (4/24) for 10 weeks
Güneri et al. [25](2003)	SD rats-acute	10 *μ*l of 1% HA vs. n 10 *μ*l of 400 g/ml EGF vs. 10 *μ*l of 2 mg/ml Mit C vs. SH	Gel	The mean closure time was 8.8 ± 1.6 days in HA-treated, 7.4 ± 1.6 days in EGF-treated, no healing in Mit C-treated for 60 days, and 15 ± 2 days in SH.
Ramalho and Bento et al. [26] (2006)	Chinchillas-subacute	EGF vs. PF vs. EGF+PF vs. DW	Gel	30.3% in EGF, 3.6% in PF, 16.5% in EGF+PF, and 8.7% in DW for 30 days
Amoils et al. [27] (1992)	Chinchilla-chronic	25 *μ*l EGF vs. 25 *μ*l PBS	Gel	81% (13/16) in EGF-treated ears vs. 25% (4/16) in PBS for 8 weeks
Lee et al. [28] (1994)	Chinchilla-chronic	50 *μ*l EGF vs. 50 *μ*l PBS	Gel	80% (12/15) in EGF and 20% (3/15) in PBS for 5 weeks
Dvorak et al. [29] (1995)	Chinchilla-chronic	50 *μ*l of EGF vs. PBS+Gel 3 times/week for 6 weeks	Gel	100% (17/17) with 3.4 weeks in EGF vs. 80% (12/15) with 3.3 weeks in PBS
Santa Maria et al. [30] (2017)	Mice-chronic	5 mg/mL HB-EGF vs. polymer only	Polymer	CSOM+ET: 100% (16/16) vs. 41% (7/17); CSOM: 100% (8/8) vs. 33.3% (3/9)

CPS: chitosan patch scaffold; SD: Sprague-Dawle; GP: guinea pigs; ET: Eustachian tube; SH: spontaneous healing; HA: hyaluronic acid; CM: collagen membrane; CBD: collagen-binding domain; HG: hydrogel; Gly: glycerol; Gel: Gelfoam: HB: heparin binding; PF: pentoxifylline; DW: distilled water; ACS: acellular collagen scaffold; PBS: phosphate buffered saline; FGF2: fibroblast growth factor-2; EGF: epidermal growth factor; TMP: tympanic membrane perforation.

**Table 2:** Summary of FGF2 and EGF effects on human acute perforation.

Authors	Study object	Study design	Size of perforation	Follow-up (months)	Treatment strategy		Closure rate		Mean closure time (days)	
					FGF2 group	Control group	FGF2 group	Control group	FGF2 group	Control group
Lou et al. [31] (2011)	Children	Retrospective control study	Medium : large = 91 : 45	6	FGF2 via GF	SH	98.5% (64/65)	85.3% (58/68)	11.1 ± 1.9	28.6 ± 3.1
Lou [32] (2012)	Adult	Randomized, controlled trial	≥50% of TM	6	FGF2 alone or via GF	SH	100% (32/32) and 97% (32/33)	55% (16/29)	12.7 ± 2.9 and 13.1 ± 3.3	46.25 ± 8.71
Zhang and Lou [33] (2012)	Adult penetrating	Prospective controlled study	<25% of TM	3	FGF2 alone	SH	100% (49/49)	77% (34/44)	12.6 ± 1.2	43.1 ± 2.5
Lou and Wang [34] (2013)	Adult	Prospective, controlled study.	>50% of TM	6	FGF2 alone	SH and EA	100% (20/20)	56% (9/16) and 60% (12/20)	12.4 ± 3.6	46.3 ± 8.7 and 48.2 ± 5.3
Lou et al. [35] (2015)	Adult-blast induced	Prospective clinical study	>75% of TM	6	FGF2 alone		94.1%(16/17)		28.4 ± 10.9	
Lou et al. [36] (2016)	Adult subacute	Prospective control study	1/8–1/2 of TM	6	FGF2 alone	SH	11/12 (91.7%)	9/17 (52.9%)	9.2 ± 2.9	18.1 ± 11.4
Lou et al. [37] (2016)	Adult	Prospective controlled study.	>25% of TM	6	FGF2 alone	GF and OFLX alone	93.2% (68/73)	85.7% (24/28) and 92.3% (36/39)	12.3 ± 8.15	14.3 ± 5.44 and 13.97 ± 8.82
Lou et al. [38] (2016)	Adults	Prospective clinical study.	>25% of TM	3	FGF2 alone	EGF alone and SH	89.3% (25/28)	86.2% (25/29) and 72.4% (21/29)	13.7 ± 7.6	12.5 ± 7.1 and 28.1 ± 12.2
Lou Z and Lou Z [39] (2017)	Adults	Randomized, controlled trial.	>25% of TM	6	FGF2 alone	EGF alone and OFLX alone	93.18% (41/44)	91.11% (41/45) and 95.65%(44/46)	10	12 and 10
Jin et al. [40] (2017)	Adults	Prospective, randomized, controlled clinical study.	>25% of TM	6	FGF2 via GF	GF vs. SH	97.9%	89.8% vs. 70.7%	15.7 ± 5.1	24.8 ± 4.9 vs. 35.7 ± 9.2 days
Lou ZC and Lou ZH [41] (2018)	Adult	Randomized, controlled trial	>25% of TM	12	FGF2	SH	95.5%	73.4%	11.9 ± 3.1	52.6 ± 18.1
Lou et al. [42] (2016)	Adult	Prospective, randomized clinical trial	≥1/8 of TM	6 months	0.1–0.15 mL of EGF	SH	91.4% (32/35)	85.2% (29/34)	8.9 ± 2.3	24.6 ± 9.7
Yang et al. [43] (2016)	Adult	Retrospective case review	≥25% of TM	6 months	0.1–0.15 mL of EGF	0.1–0.15 mL of OFLX and SH	93.5% (29/31)	93.2% (41/44) and 82.2% (37/45)	12.9 ± 5.3	13.3 ± 4.9 and 35.7 ± 9.2
Lou ZC and Lou Z [44] (2018)	Adult	Prospective, randomized, controlled	≥50% of TM	6 months	0.1–0.15 mL of EGF	Gelatin patch and SH	97.8%	86.7% and 82.2%	11.12 ± 4.60	13.67 ± 5.37 and 25.65 ± 13.32
Lou et al. [45] (2019)	Adult subacute	Prospective study	≥1/8 of TM	6 months	0.1–0.15 mL of EGF	SH	96.2%	61.1%	9.1 ± 3.9	20.6 ± 10.7
Lou [46] (2019)	Adult chronic traumatic	Case observation study		6 months	0.1–0.15 mL of EGF		100% (24/24)		6.1 ± 2.3 days	

SH: spontaneous healing; OFLX: ofloxacin drops; FGF2: fibroblast growth factor-2; EGF: epidermal growth factor; GF: Gelfoam; EA: edge approximation; TM: tympanic membrane.

**Table 3:** Summary of FGF2 and EGF effects on human chronic perforation.

Authors	Study object	Etiology	Study design	Size of perforation		Follow-up (months)	Treatment strategy		Closure rate		Closure time (weeks)	
				FGF2 group	Control group		FGF2 group	Control group	FGF2 group	Control group	FGF2 group	Control group
Hakuba et al. [47] (2003)	14 adult	COM	Control study	16.5%	9.6%	3	FAS for 2 weeks	SAS for 2 weeks	100% (9/9)	40% (2/5)	3.7 (2-6)	3.6 (2-4)
Hakuba et al. [48] (2010)	87 adult	60 COM:7 VT:20 trauma	Observation study	14.4%		3	FAS for 3 weeks		92.0% (80/87)		4.8	
Kanemaru et al. [49] (2011)	63 adult	COM	Randomized control trial.	2/3 : >2/3 = 34 : 19	<2/3 : >2/3 = 8 : 2	3	FGF via GF for 3 weeks	Saline via GF	98.1% (52/53)	10.0% (1/10)	41 (78.8%) within 3	12
Hakuba et al. [50] (2013)	116 adult	77 COM:12 trauma:15 VT	Observation study	50% : ≥50% = 98 : 18		12	FAS for 3 weeks		62% (73/116)		Unclear	
Acharya et al. [51] (2015)	12 children	VT or COM	Prospective cohort study	6-40%		12	FGF via Gel for 3 weeks		58% (7/12)		2-12	
Hakuba et al. [52] (2015)	153 adult	COM	Retrospective cohort study	25% : 25 − 50% : >50% = 73 : 55 : 25		12	FAS for 3 weeks		66.0% (101/153)		4.5	
Omae et al. [53] (2017)	10 adult	5 COM:trauma	Prospective clinical trial	≤1/3 : 1/3 − 2/3 = 8 : 2		3	FGF via Gel for 4 weeks		88.9% (8/9)		57 days	
Santos et al. [54] (2020)	54 adult	Trauma, COM, and unknown	Randomized controlled trial	82.4%	75	3	FGF via Gel for 3 weeks	SGF for 3 weeks	57.5%(23/40)	71.4% (10/14)	14 in one, 8 in 2, and 1 in 3	4 in one, 5 in 2, and 1 in 3
Ramsay et al. [55] (1995)	17 adult	1 traumatic and 16 COM	Double-blind randomized control trial	Unclear	Unclear	2.6	EGF via paper	PBS+paper	0/8, size of perforation mean decrease 0.3%	1/9, 2.7%		

FAS: bFGF via atelocollagen and sealed by silicon layer; SAS: saline via atelocollagen and sealed by silicon layer; FGF: FGF-2 via gelatin sponge and sealed by fibrin glue; GF: gelatin sponge and fibrin glue. COM: chronic otitis media; VT: ventilation tube; Gel: gelatin sponge.

**Table 4:** The dose- and starting time-dependent effect of growth factors on the eardrum healing.

Authors	Study object	Study design	Size of perforation	Follow-up (months)	Growth factors	Dosage	Application time	Closure rate	Mean closure time (days)
Mondain et al. [60] (1991)	Sprague Dawley rats-acute				FGF-2	2000 ng, 400 ng or 200 ng vs. saline		100% with 3.16 days in 2000 ng FGF2; 12/15 with 6.1 days in 400 ng; 9/15 with 6.3 days in 200 ng; 3/15 with 8.86 days in saline alone	
Lou et al. [35] (2015)	Human-acute	Prospective clinical study	25% of TM	6 months	FGF-2		The durations of ≤3 vs. >3 days	96.6% (28/29) vs. 100% (17/17)	17.5 ± 5.1 vs. 8.5 ± 2.1
Lou et al. [66] (2014)	Human-acute	Prospective clinical study	25% of TM	3 months	FGF-2	0.1-0.15 ml vs. 0.25-0.3 ml		92% vs. 100%	11.8 ± 4.7 vs. 15.1 ± 6.1
Lou et al. [45] (2019)	Human-acute	Prospective clinical study	25% of TM	6 months	EGF	0.1-0.15 ml vs. 0.25-0.3 ml		93.3% vs. 89.1%	10.20 ± 5.13 vs. 14.39 ± 6.20
							The durations of ≤3 vs. >3 days	90.2% vs. 93.3%	13.15 ± 5.80 vs. 11.25 ± 7.15

FGF2: fibroblast growth factor-2; EGF: epidermal growth factor; TM: tympanic membrane.

## Data Availability

All data generated or analysed during this study are included in this published article.
